# Pharmacological inhibition of host cell neddylation reduces intoxication of cells by diphtheria toxin and clostridial enterotoxins TcdB and C2

**DOI:** 10.1128/spectrum.03872-25

**Published:** 2026-04-02

**Authors:** Irina König, Katharina Oßwald, Lisa Schneider, Holger Barth, Panagiotis Papatheodorou

**Affiliations:** 1 Institute of Experimental and Clinical Pharmacology, Toxicology and Pharmacology of Natural Products, Ulm University Medical Center, Ulm, Germany; Shandong First Medical University, Jinan, Shandong, China

**Keywords:** neddylation, bacterial toxin, toxin uptake, endocytosis, inhibitor

## Abstract

NEDD8 is a ubiquitin-like molecule that is conjugated to lysine residues of protein substrates in a process known as neddylation, which affects their stability, degradation, and interactions. It was recently found that the neddylation inhibitor MLN4924 prevents endocytic uptake of herpes simplex virus (HSV) into target cells, implicating a broader role for neddylation in viral entry and infection. Because AB-type bacterial toxins share similar cell entry mechanisms with viruses, we studied the impact of neddylation in the cellular internalization of a prototypical, well-established model of bacterial AB-type toxins, namely the diphtheria toxin (DT). We found that pretreatment of HeLa cells with MLN4924 reduced morphological changes and protein biosynthesis inhibition induced by DT. Arctigenin, another neddylation inhibitor acting at a later step of the neddylation cascade, was also capable of reducing DT intoxication in HeLa cells. In combination, both neddylation inhibitors synergistically increased their inhibitory potential against DT. Further mechanistic studies with MLN4924 revealed that the neddylation inhibitor neither interfered with cell surface binding, proteolytic activation, enzymatic activity, and membrane translocation of DT, nor with endosomal acidification in HeLa cells. Finally, we could demonstrate that pharmacological inhibition of neddylation also affects further bacterial protein toxins, namely single-chain toxin *Clostridioides difficile* TcdB and binary toxin *Clostridium botulinum* C2. In conclusion, our study is the first to support a connection between neddylation and the cellular uptake of bacterial AB-type toxins, thereby establishing a foundation for future investigations into these molecular mechanisms and for the development of anti-toxin strategies.

**IMPORTANCE:**

Neddylation, a ubiquitin-like protein modification, is emerging as a critical regulator of viral entry into cells. Here, we demonstrate for the first time that pharmacological inhibition of neddylation impairs the intoxication of target cells with diverse bacterial AB-type toxins. Mechanistic studies suggest that neddylation most likely reduces toxin internalization, thus supporting the connection between neddylation and endocytic processes. Our study offers new opportunities to explore anti-toxin interventions and highlights the use of bacterial toxins as molecular probes to further study the role of neddylation in endocytic trafficking.

## INTRODUCTION

Neddylation refers to a post-translational modification (PTM) in which the ubiquitin-like molecule neural precursor cell expressed, developmentally downregulated 8 (NEDD8) is covalently attached to a lysine residue of target proteins, thereby influencing either their stability, subcellular distribution, and/or binding to other molecules (1). The process of neddylation resembles the ubiquitylation cascade but relies on its own enzymes for activation (E1; NAE, NEDD8-activating enzyme), conjugation (E2; UBE2M/UBC12 or UBE2F, NEDD8-conjugating enzymes), and ligation (E3; numerous NEDD8 ligases), respectively (2). The cullin family represents the most extensively characterized group of proteins that undergo neddylation. Cullins act as scaffolds in the assembly of multi-subunit E3 ubiquitin ligase complexes known as Cullin-RING Ligases (CRLs) (3). However, several non-cullin substrates have been identified, indicating a plethora of processes potentially controlled by neddylation (4).

A recent study has demonstrated a critical role for neddylation in the cell entry of alphaherpesviruses, such as herpes simplex virus 1 (HSV-1) (5). Prompted by these findings and the fact that viruses often share common cell entry strategies with bacterial protein toxins, we were asking whether neddylation plays a pivotal role in the cellular uptake of bacterial toxins, such as diphtheria toxin (DT), a prototypical, well-studied AB-type exotoxin secreted mainly by *Corynebacterium diphtheriae* (6). DT is a single-chain protein toxin consisting of two major subunits, which enters into host cells via receptor-mediated endocytosis upon binding to its cell surface receptor HB-EGF (7). Within acidified endosomes, the binding and translocation subunit DTB forms translocation pores for the delivery of the enzymatically active DTA subunit into the cytosol (8–10). Eventually, DTA leads to the inhibition of protein synthesis and apoptosis in target cells by mono-ADP-ribosylation of the elongation factor 2 (EF-2) (11, 12).

DT-induced cell rounding in HeLa cells provides a specific and ideal endpoint for microscopically observing DT uptake and subsequent cellular intoxication (13). Thus, we tested the role of neddylation during DT intoxication on HeLa cells with well-established pharmacological neddylation inhibitors, such as MLN4924 (14) and arctigenin, a more recently identified neddylation inhibitor acting at a later step of the neddylation cascade than MLN4924 (15). Importantly, we can show here with both inhibitory compounds and by implementing various experimental approaches that the pharmacological inhibition of neddylation delays DT intoxication in HeLa cells. Further mechanistic analysis revealed that MLN4924 neither interfered with cell surface binding, proteolytic activation, ADP-ribosyltransferase activity, and membrane translocation of DT, nor with endosomal acidification in HeLa cells. Finally, we could demonstrate that pharmacological inhibition of neddylation also reduces cytotoxicity of two other bacterial protein toxins, namely single-chain toxin *Clostridioides difficile* TcdB and binary toxin *Clostridium botulinum* C2.

Thus, our study links bacterial toxin uptake to neddylation, suggesting a new therapeutic approach for toxin-mediated diseases by targeting host neddylation pathways.

## RESULTS

### MLN4924 reduces DT intoxication of HeLa cells

To test whether neddylation interferes with DT intoxication of HeLa cells, we initiated our investigations using the well-established neddylation inhibitor MLN4924 (14). First, it was necessary to identify the maximum tolerable concentration of MLN4924 for HeLa cells. To this end, HeLa cells were incubated with increasing concentrations (up to 40 µM) of MLN4924, followed by microscopic analysis of cell morphology and MTT-based measurements of the cell viability. All tested MLN4924 concentrations neither changed the cell morphology (Fig. S1A) nor decreased the cell viability of HeLa cells over the tested incubation period of 5 h (Fig. S1B). Based on these findings, we decided to proceed with 40 µM MLN4924 in subsequent experiments. This concentration was chosen as it demonstrated that cell viability was not decreased within the 5-h treatment window, a timeframe sufficient for assessing DT-induced toxicity in HeLa cells.

**Fig 1:**
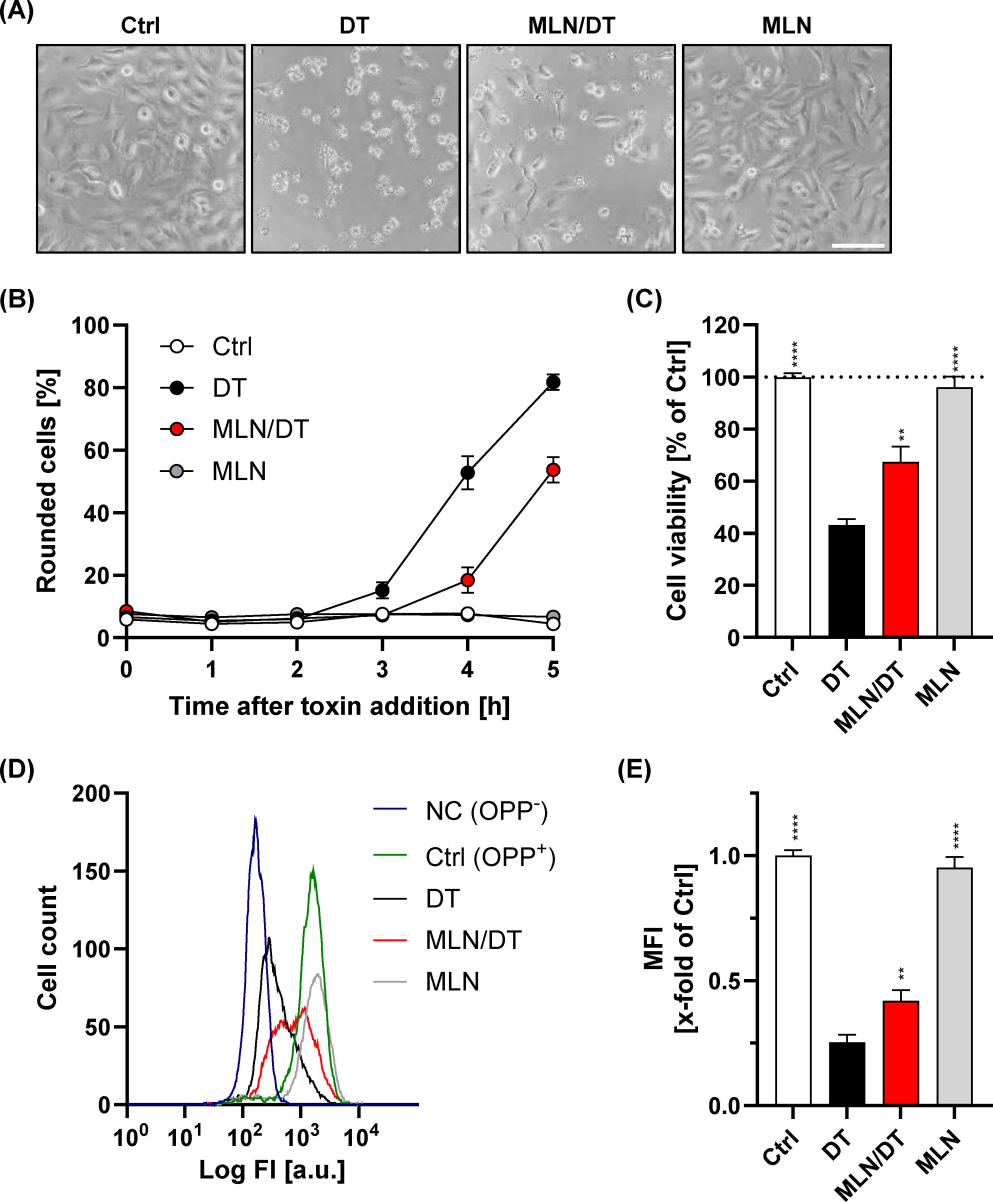
MLN4924 reduces DT-mediated intoxication of HeLa cells. (**A**) Analysis of morphological changes induced by DT. Representative microscopic images of HeLa cells either preincubated for 20 min with 40 µM MLN4924 (MLN/DT) or with solvent only (DT), followed by 5 h intoxication with DT (2 nM). Ctrl, control HeLa cells without any treatment; MLN, HeLa cells preincubated only with 40 µM MLN4924. Scale bar represents 100 µm. (**B**) Quantification of cell rounding over time of the experiment shown in (**A**). Shown are the mean percentage values of round cells (in % from total cells), as calculated from three independent experiments performed in duplicate or triplicate. Error bars represent ±SEM (*n* = 8). (**C**) Analysis of cell viability by MTS test following 5 h incubation with DT. Absorbance was measured for each condition at 490 nm and normalized to the absorbance values of control cells without any treatment (Ctrl). A bar diagram represents relative cell viability (in % from control) as calculated from three independent experiments performed with triplicates. Error bars represent ±SEM (*n* = 9). Significance was determined between the DT only condition (DT) and all other conditions (**P < 0.01 and ****P < 0.0001). (**D**) Effect of MLN4924 on protein synthesis in DT-treated cells. Flow cytometry-based analysis of OPP incorporation in cells following DT intoxication. A representative histogram of all conditions is shown. Cells were treated as indicated either with 40 µM MLN4924 (MLN), 750 pM DT (DT), the combination of MLN4924 and DT (MLN/DT), or were left untreated (Ctrl (OPP+)). Conditions containing MLN4924 were preincubated for 20 min at 37°C with the component prior to DT addition. After 90 min of intoxication, 5 µM OPP were added to all samples except for a negative control (NC (OPP−)) representing autofluorescence of the cells. Each measurement was performed with 10,000 cells. (**E**) Quantitative analysis of (**D**). Shown is the median fluorescence intensity of cells *x*-fold to the untreated control (Ctrl), which was set to 1. Values are given as mean ± SEM of three independent assays with triplicates (*n* = 9). Significance was determined between the DT only condition (DT) and all other conditions (**P < 0.01 and ****P < 0.0001).

Next, HeLa cells were preincubated with MLN4924 for 20 min, followed by addition of DT. Cell morphology was then monitored by microscopy every 1 h for up to 5 h. Following this approach, we found that DT-induced cell rounding was markedly reduced in MLN4924-preincubated HeLa cells after 5 h of DT intoxication when compared to cells preincubated with solvent only (Fig. 1A). Notably, MLN4924 itself did not induce any cell rounding after the 5-h time period. Quantification of DT-induced cell rounding over time revealed a delay of intoxication in MLN4924-preincubated HeLa cells (Fig. 1B). Consistently, the decrease in cell viability by DT after 5 h of intoxication was less pronounced in MLN4924-preincubated HeLa cells, when compared to untreated HeLa cells (Fig. 1C).

We then aimed to confirm these findings with a more specific, fluorescence- and flow cytometry-based readout of DT intoxication. To this end, we applied O-propargyl-puromycin (OPP) labeling to assess total protein synthesis in DT-intoxicated HeLa cells that were preincubated for 20 min either with or without MLN4924. As a result, we found that preincubation with MLN4924 significantly inhibited the decrease in protein synthesis observed in HeLa cells after intoxication with DT for 90 min (Fig. 1D and E). Notably, using the same experimental approach, MLN4924 preincubation also inhibited DT-mediated protein synthesis inhibition in Vero cells (Fig. S2A and B).

### The distinct neddylation inhibitor arctigenin is also capable of reducing DT intoxication of HeLa cells

To further validate these findings with a distinct inhibitor of neddylation, we subsequently examined arctigenin. Unlike MLN4924, which inhibits the first step in the neddylation cascade by targeting the NEDD8-activating enzyme (NAE), arctigenin specifically targets and inhibits the activity of the NEDD8-conjugating enzyme UBC12 (15). In order to identify the maximum tolerable concentration of arctigenin for HeLa cells, we incubated the cells with increasing concentrations (up to 160 µM) of arctigenin, prior to microscopic analysis of cell morphology and MTT-based measurements of the cell viability. All tested arctigenin concentrations did not induce changes in cell morphology (Fig. S3A) and cell viability was only slightly reduced over the tested incubation period of 5 h (Fig. S3B). Based on these findings, we decided to use 160 µM arctigenin in subsequent experiments.

**Fig 2:**
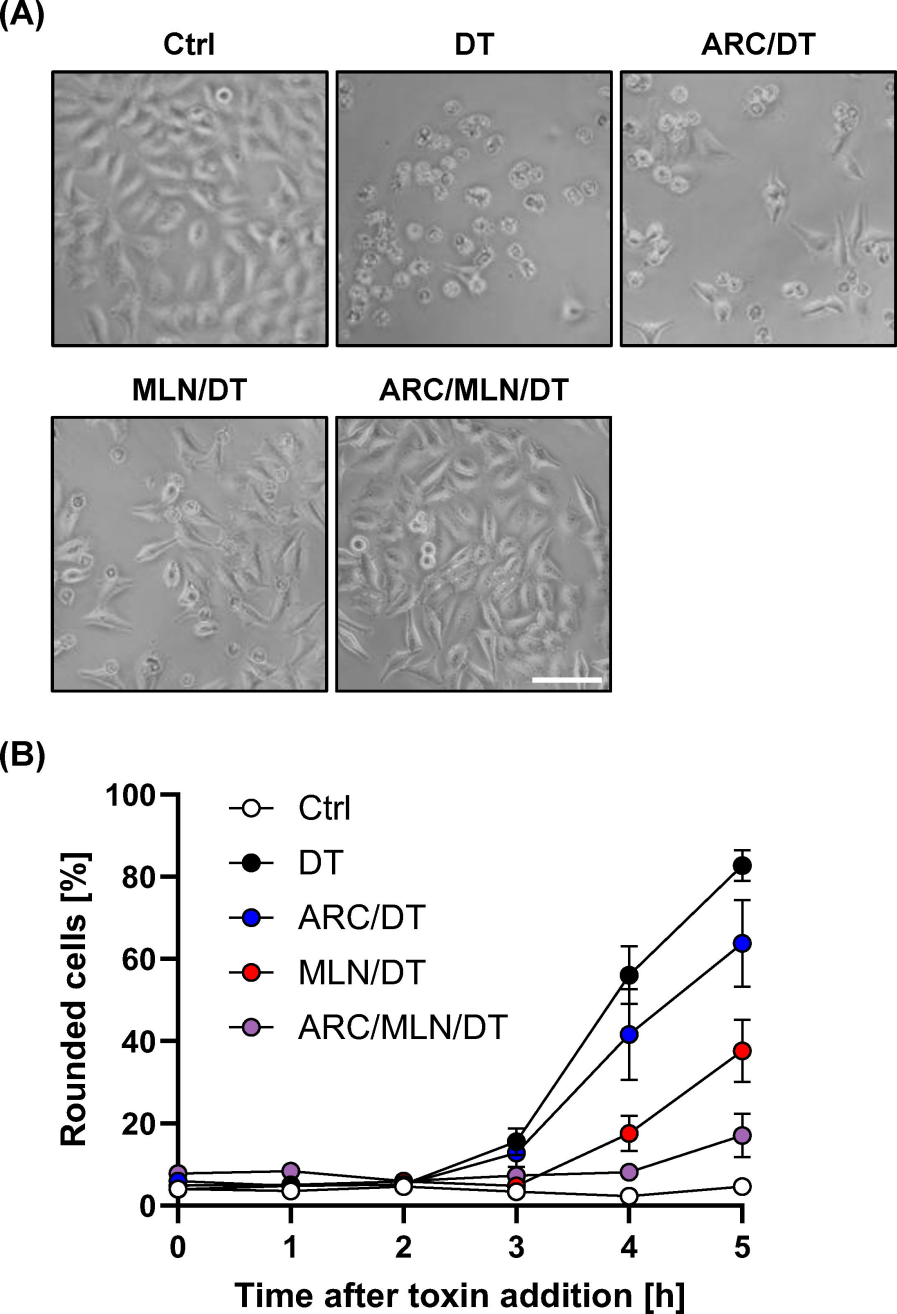
Arctigenin and MLN4924 synergistically inhibit DT intoxication of HeLa cells. (**A**) Analysis of DT-induced cell rounding. Representative microscopic images of HeLa cells after 5 h DT (1 nM) intoxication. Cells were either preincubated for 20 min with 40 µM MLN4924 (MLN/DT), 160 µM arctigenin (ARC/DT), or the combination of both inhibitors (ARC/MLN/DT). Untreated cells (Ctrl) and cells treated with DT only (DT) served as controls. Scale bar (white) represents 100 µm. The percentage of rounded cells is given as mean ± SEM values (% of total cells), based on three independent experiments performed with duplicates or triplicates (*n* = 8). Data are shown as a time course (**B**).

To assess the inhibitory potential of arctigenin against DT, HeLa cells were preincubated for 20 min with arctigenin or were left untreated, followed by intoxication with DT and microscopic analysis of cell morphology over a time period of up to 5 h. In accordance with the results obtained above with MLN4924, pharmacological inhibition of neddylation with arctigenin also reduced DT-induced cell rounding in HeLa cells. Furthermore, we could show in the same experimental setting that arctigenin in combination with MLN4924 synergistically enhanced the reduction in DT-induced cell rounding compared to either inhibitor alone (Fig. 2A and B). These findings suggest a synergistic effect of both distinct neddylation inhibitors and further support a role of neddylation during the intoxication process of DT.

Eventually, we assessed the ability of MLN4924 and arctigenin to inhibit neddylation in HeLa cells. To this end, we chose to analyze the neddylation status of Cullin-1 in whole-cell lysates from HeLa cells preincubated for 20 min or 24 h with MLN4924 and arctigenin, respectively. Cullin subunits of Cullin-RING ligases (CRLs), including Cullin-1, are the main target of neddylation (16). Immunoblotting analysis using a Cullin-1-specific antibody revealed a significant decrease in the levels of NEDD8-conjugated Cullin-1 in cells treated for 24 h with either compound compared to untreated controls (Fig. S4). Cullin-1 neddylation was completely absent also after 20 min preincubation with MLN4924. Thus, our data confirm that both substances inhibit neddylation in HeLa cells. This finding, combined with their synergistic inhibition of DT intoxication, suggests a potential correlation between neddylation and cellular susceptibility to DT.

### Studies on the DT-inhibiting mode of action of MLN4924

Next, we investigated the underlying inhibitory mechanism of MLN4924 against DT in more detail. A previous study with viruses suggested an inhibitory role of MLN4924 on endocytosis (5). Thus, it is conceivable that MLN4924 not only inhibits the endocytosis of DT but also that of other bacterial toxins. Alternatively, MLN4924 might interfere directly with DT or with important steps during cell entry of the toxin, such as binding or proteolytic activation at the host cell membrane and membrane translocation, respectively, for the delivery of its enzyme domain from endosomes into the host cell cytosol.

**Fig 3:**
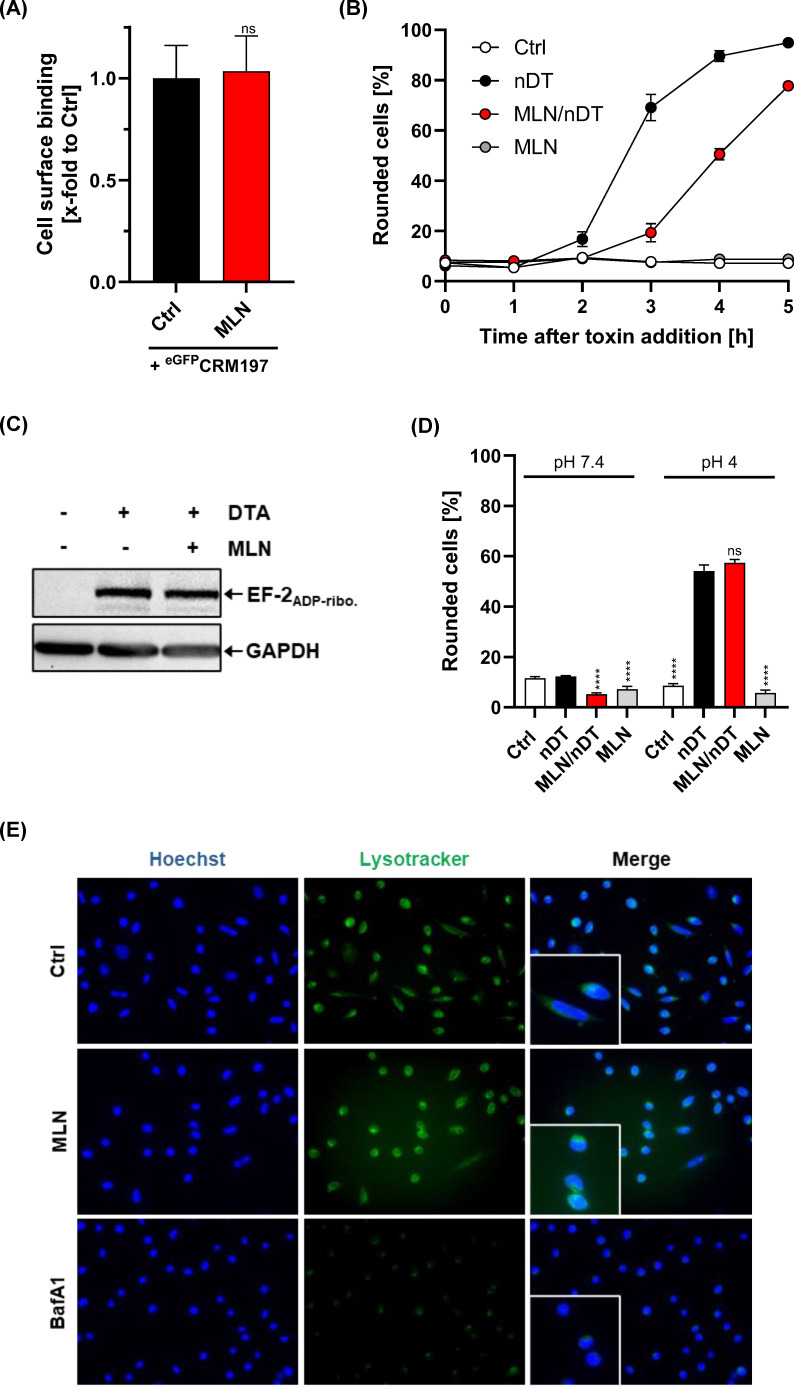
Analysis of the DT-inhibiting mode of action of MLN4924. (**A**) Flow-cytometric analysis of the effect of MLN4924 on DT binding DT to cells (enzymatically inactive DT variant ^eGFP^CRM197 was used). Cells were detached with EDTA and pretreated with 40 µM MLN4924 for 20 min, followed by incubation with 250 nM ^eGFP^CRM197 on ice for 30 min. Increase in fluorescence was measured from 10,000 cells and is shown as cell surface binding of ^eGFP^CRM197 *x*-fold to the control (Ctrl), which was set to 1. Values are given as mean ± SEM of three independent assays with triplicates (*n* = 9). Significance was determined between the ^eGFP^CRM197 only condition (Ctrl) and the MLN4924 pretreated condition (ns = not significant). (**B**) The impact of MLN4924 on the activation of DT on the cell surface was investigated by intoxication of cells with nicked DT (nDT). For this, HeLa cells were preincubated at 37°C for 20 min in medium either without or with 40 µM MLN4924, prior to intoxication for 5 h at 37°C with 10 nM nDT. Untreated cells (Ctrl) and cells treated only with MLN4924 (MLN) served as controls. Shown is the mean percentage of rounded cells (% of total) ±SEM, calculated from three independent experiments performed in triplicates (*n* = 9). (**C**) Effect of MLN4924 on the enzyme activity of DT *in vitro*. The enzyme activity was detected by ADP ribosylation of elongation factor 2 (EF2_ADP-ribo_.) by the enzyme domain of DT (DTA). Therefore, HeLa cell lysates were incubated with 40 µM MLN4924 (MLN), 5 pM DTA, and 1 µM biotin-labeled NAD^+^ at 37°C for 30 min. After SDS-PAGE, the ADP-ribosylated EF2 was detected by Western blotting. GAPDH serves for loading control. (**D**) Analysis of pH-driven DT translocation across cell membranes in the absence and presence of MLN4924. HeLa cells were pretreated for 20 min with 100 nM BafA1 ±40 µM MLN4924 (MLN), followed by binding of 50 nM nDT for 30 min on ice. Translocation was triggered by adding MEM at pH 4. Untreated cells served as control (Ctrl). To confirm pH-dependent translocation of nDT, equally treated cells without pH drop (pH 7.4) were included. Images were taken 4 h after the acidic pulse, and the percentage of rounded cells was quantified from six images per condition, obtained from triplicate wells with two images captured per well. Values are given as mean ± SEM from two independent experiments (*n* = 12). Statistical analysis was performed between each condition and the respective nDT approach at pH 7.4 or 4 (ns *P* ≥ 0.05, ****P < 0.0001). (**E**) Endosomal acidification in MLN4924-treated cells was evaluated using the dye Lysotracker and fluorescence microscopy. For this, HeLa cells were treated either with 40 µM MLN (MLN) for 20 min or with 200 nM Bafilomycin A1 (BafA1) for 1 h at 37°C. Untreated cells (Ctrl) served as control. Immunofluorescence staining was performed, where nuclei (Hoechst) and acidic cell compartments (Lysotracker) were stained. Shown are representative images of the individual channels and their merge.

First, we tested via flow cytometry the effect of MLN4924 preincubation on the binding of an enzymatically inactive DT variant (termed CRM197), fused to enhanced GFP (^eGFP^CRM197), to HeLa cells. Importantly, binding of ^eGFP^CRM197 was not reduced on MLN4924-preincubated HeLa cells when compared to cells without MLN4924 preincubation (Fig. 3A). Then, we tested whether a nicked DT variant (termed nDT), which does not require further proteolytical activation by host proteases at the cell surface, is inhibited by MLN4924. Intriguingly, cell rounding induced by nDT on HeLa cells was inhibited by preincubation of the cells with MLN4924 (Fig. 3B). Thus, from these experiments, we concluded that the MLN4924 compound seems not to interfere with binding and/or activation of DT on the cell surface of host cells.

Next, we tested whether MLN4924 acts as a direct inhibitor of the enzyme domain of DT. For that purpose, lysates obtained from HeLa cells were incubated with the enzyme domain of DT (DTA), either in the presence or absence of MLN4924. Biotinylated NAD was used as co-substrate in these *in vitro* reactions, since it allows for the visualization of ADP-ribosylated proteins via streptavidin-HRP (horseradish peroxidase) and enhanced chemiluminescence (ECL) after SDS-PAGE and immunoblotting. By that approach, we observed that the presence of MLN4924 did not inhibit ADP-ribosylation of the host target protein EF-2 (elongation factor 2) by DTA (Fig. 3C).

To investigate whether MLN4924 directly interferes with the membrane translocation step of DT during cell entry, we employed intoxication conditions with nicked DT (nDT) in which receptor-mediated endocytosis is circumvented. Specifically, HeLa cells were subjected to an ‘acidic pulse’ that drives surface-bound nDT to insert into the plasma membrane and translocate its DTA domain directly from the plasma membrane into the cytosol, thereby bypassing the endosomal trafficking route required for substrate modification. Bafilomycin A1 (BafA1) was included in this experiment to inhibit endosomal acidification and thereby prevent any contribution of endosomal translocation to intoxication. Notably, preincubation with MLN4924 did not diminish nDT-induced cell rounding (Fig. 3D), indicating that the compound does not affect the membrane translocation step of DT.

Translocation of DTA from endosomes into the cytosol depends on endosomal acidification, which triggers conformational changes within the translocation domain of DT. We therefore investigated whether MLN4924 reduces endosomal acidification, potentially explaining its inhibitory mode of action against the toxin. Surprisingly, preincubation of HeLa cells with MLN4924 did not impair endosomal acidification, as assessed by Lysotracker Green staining and fluorescence microscopy (Fig. 3E).

### MLN4924 inhibits single-chain and binary AB-type toxins

We next sought to investigate the broader role of neddylation in bacterial toxin internalization and to determine whether this post-translational modification regulates fundamental cellular processes involved in toxin uptake. DT is produced and released by the bacteria as a single-chain AB-type toxin, which belongs to the “short-trip toxins” that deliver their enzyme domains into the cytosol directly from endosomes after cell entry. Other well-studied members of this group are the single-chain toxin *C. difficile* TcdB (formerly toxin B) and the binary *C. botulinum* C2 toxin. We therefore investigated these toxins to understand the significance of neddylation for their cellular uptake and/or toxic effects on target cells.

**Fig 4:**
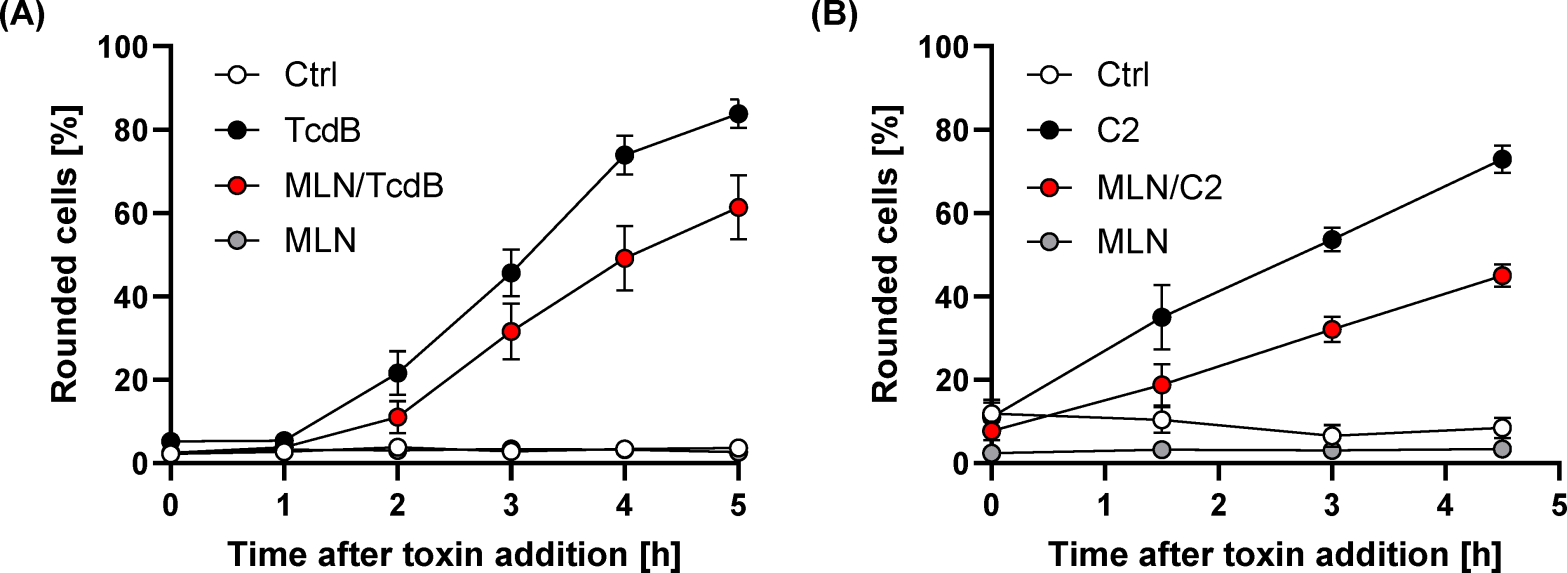
Effect of MLN4924 on the intoxication of HeLa cells by TcdB or C2 toxin. HeLa cells were preincubated at 37°C for 20 min in medium either without or with 40 µM MLN4924, prior to incubation for 5 h at 37°C with (**A**) TcdB (8 pM; TcdB vs MLN/TcdB) or (**B**) C2 toxin (2 nM C2I plus 3.32 nM C2IIa; C2 vs MLN/C2). Untreated cells (Ctrl) and cells treated only with MLN4924 (MLN) served as controls. (**A** and **C**) show the time-course quantification of the mean percentage of rounded cells (in % from total cells). Data were calculated from three independent experiments performed in triplicate. Error bars represent ±SEM (*n* = 9).

Since both TcdB and C2 toxin induce the collapse of the cytoskeleton, which results in cell rounding, we used this sensitive and specific endpoint to investigate the impact of MLN49284 on both toxins. To this end, HeLa cells were preincubated with MLN4924 for 20 min, followed by intoxication with either TcdB or C2 toxin. Microscopic images were taken for up to 5 h, and the percentage of rounded cells from the total cells was determined. Interestingly, cell rounding after intoxication with TcdB (Fig. 4A and B) or C2 (Fig. 4C and D) was significantly reduced in MLN4924-preincubated HeLa cells in comparison to cells without preincubation with the compound.

## DISCUSSION

Our study is the first to reveal a connection between neddylation and the cellular uptake of bacterial protein toxins. This conclusion is supported by data that we obtained from three distinct toxins that utilize different host receptors for cell entry, as well as from two pharmacological inhibitors targeting separate steps of the neddylation cascade. We further confirmed MLN4924-mediated inhibition of DT intoxication in Vero cells using a quantitative OPP assay. Importantly, MLN4924 neither interfered with cell-surface binding, proteolytic activation, ADP-ribosyltransferase activity, and membrane translocation of DT, nor with endosomal acidification in HeLa cells, supporting a broader, more general role of neddylation in endocytic processes. In line with this, since many viruses share endocytic pathways with bacterial toxins, neddylation was recently shown to play a critical role also in the cellular uptake of certain viruses (5).

Ubiquitination and neddylation are closely related post-translational modification pathways that involve the covalent attachment of the ubiquitin (Ub) protein and the ubiquitin-like protein NEDD8, respectively, to target proteins (17). Typically, ubiquitination marks thousands of proteins across all cellular compartments for proteasomal degradation. However, for cell-surface proteins, ubiquitin serves as a signal for clathrin-dependent internalization and/or sorting to lysosomes for degradation (18). Ubiquitination involves cullin-based ubiquitin ligases which in turn are activated by neddylation of the cullin subunits (19), the main target proteins of neddylation (20). Therefore, pharmacological inhibition of neddylation would negatively impact also the ubiquitination of cell-surface receptors and inhibit in an indirect manner their internalization. Strikingly, there is increasing evidence that neddylation is also directly involved in the endocytosis of membrane receptors, for example, as the EGF and TGF-β receptors (21, 22). Thus, we hypothesize that neddylation inhibition affects the internalization of cell-surface receptors utilized by bacterial toxins for cell entry.

Of interest is the fact that certain pathogenic bacteria, such as enteropathogenic or enterohemorrhagic *Escherichia coli*, inject a cycle-inhibiting factor (Cif) into host cells through the type III secretion system (T3SS), which manipulates host ubiquitin-dependent proteasomal degradation by interfering with NEDD8-conjugated cullin-based ligases (23). Given that clathrin-mediated endocytosis and the internalization of many cell-surface receptors depend on proper ubiquitination regulated by cullin-based ubiquitin ligases, and that the activation of these ligases is controlled by neddylation of cullin scaffolds, it would be compelling to investigate whether ectopic Cif expression in human cells reduces their susceptibility to certain viruses and/or bacterial toxins, potentially by inhibiting the neddylation-dependent internalization of receptors that these viruses and/or bacterial toxins use for cell entry.

NEDD8 was found overexpressed in several cancers (24, 25), and for that reason, protein neddylation became a promising target for cancer therapy (26–28). Inhibitors of this pathway, such as MLN4924, have proven especially promising. MLN4924 selectively inhibits the NEDD8-activating enzyme (NAE) (2) and exerts antitumor activity by inducing cell-associated autophagy, apoptosis, and senescence (29, 30). Consequently, MLN4924 has been widely acknowledged as a potent anti-cancer drug and investigated as pevonedistat in a series of I/II/III clinical trials for solid and non-solid cancers (31). Accordingly, arctigenin has also attracted growing attention due to its antitumor capabilities (32, 33). Given the fact that both neddylation inhibitors used in our study, MLN4924 and arctigenin, are already used in clinical trials, we propose to consider these compounds also as anti-toxin therapeutics, while their anti-toxin applications remain to be fully elucidated. Notably, both compounds exhibit secondary pharmacological actions that warrant consideration, as they may influence the occurrence of adverse reactions during therapy (34, 35).

Whereas “short-trip” toxins (DT, TcdB, and C2) deliver their enzyme subunits directly from acidified endosomal vesicles into the cytosol, “long-trip” toxins, such as cholera, pertussis, and Shiga toxin, deliver their enzymatic domains from the ER into the cytosol after retrograde transport through the endomembrane system including the Golgi apparatus (36). Both types of toxins require binding to cell-surface receptors for initiating receptor-mediated endocytosis. Notably, “long-trip” toxins usually bind to carbohydrate-based receptors like glycolipids or to sialic acid-containing glycoproteins. One would assume that the uptake of “long-trip” toxins is not affected by neddylation inhibitors because their uptake does not depend on the ubiquitination and/or neddylation of cell-surface proteins. The “short-trip” toxins DT and TcdB use proteinaceous receptors for cell entry (7, 37). Although C2 toxin was found to bind to certain carbohydrates at the cell surface (38), it remains unclear whether carbohydrates are sufficient for C2 binding and cell entry or whether a proteinaceous receptor is additionally required.

The cellular uptake of bacterial protein toxins is well-defined, including several consecutive steps. Moreover, many bacterial toxins exhibit *in vitro* clear cytopathic effects on cultured cell monolayers (e.g., cell rounding) which can be easily monitored and quantified. For that reason, we suggest that bacterial toxins might be used in the future as valuable tools for studying the role of neddylation in endocytosis in more detail. Conversely, neddylation inhibitors can help uncover the molecular mechanisms underlying toxin receptor function.

## MATERIALS AND METHODS

### Compounds

The unnicked diphtheria toxin from *Corynebacterium diphtheriae* used in all experiments was obtained from Merck, Germany (cat. no. 322326-1MG). TcdB toxin from *Clostridioides difficile* was generously provided by Klaus Aktories (University of Freiburg, Germany). C2 toxin from *Clostridium botulinum* was expressed and purified as described elsewhere (39). MLN4924 and arctigenin were acquired commercially from Bio-Techne, Germany (cat. no. 6499) and Biomol, Germany (cat. no. Cay14913-10), respectively.

### Cell culture

HeLa or Vero cells were cultured in minimal essential medium (MEM; Gibco-Life Technologies, Carlsbad, CA, USA) supplemented with 10% fetal calf serum (Gibco-Life Technologies), 0.1 mM MEM-non essential amino acids (MEM-NEAA; Gibco-Life Technologies), 2 mM l-Glutamine (PAN-BIOTECH, Aidenbach, GER), 1 mM sodium pyruvate (Gibco-Life Technologies), and 1% (100 U/mL) penicillin-streptomycin (Gibco-Life Technologies) at 37°C, 5% CO_2_ with constant humidity. The cells were sub-cultivated every 3–4 days in a split ratio of 1:3 or 1:10 after trypsinization.

### Cytotoxicity and cell viability assay

For cytotoxicity and cell viability testing, 7.8 × 10^3^ cells/well were seeded in 96-well plates and incubated for 24 h at 37°C. Cultivation medium was removed, and the cells were treated with fresh serum-free medium (100 µL/well) containing the components specified in each experiment. Conditions involving MLN4924 or arctigenin were pretreated with the compounds for 20 min prior to intoxication (37°C; 5% CO_2_). Intoxication with DT/nDT, TcdB, and C2 leads to cytotoxic effects indicated by morphological changes in the cells (cell rounding). These morphological changes were documented with a Leica MC170 HD camera (Leica Microsystems Ltd., Heerbrugg, Switzerland) connected to Leica DMi1 light microscope (Leica Microsystems CMS GmbH, Wetzlar, Germany). The percentage of rounded cells out of the total amount of cells in each image was quantified manually. Cell viability was determined using the CellTiter 96 AQueous One Solution Cell Proliferation Assay (MTS assay; Promega GmbH, Walldorf, Germany), according to the manufacturer’s protocol. As a positive control for reduced viability, the cells were treated with 20% (vol/vol) dimethyl sulfoxide (DMSO; Carl Roth, Karlsruhe, Germany).

### OPP-based protein synthesis assay

For the cell-based protein synthesis assay, HeLa or Vero cells (1 × 10^5^ cells/well) were seeded in 24-well plates and incubated for 24 h (37°C; 5% CO_2_). The cells were treated with MLN4924, DT, or a combination of both, as indicated. Untreated cells served as a control for maximal OPP incorporation, while cells incubated without OPP served as a negative control for background fluorescence. Protein synthesis was assessed using the Click-&-Go Plus OPP Protein Synthesis Assay Kit (Click Chemistry Tools), following the manufacturer’s instructions with modifications as described previously (40). In brief, HeLa cells were labeled with 5 µM OPP for 30 min and Vero cells with 30 µM OPP for 1 h (37°C; 5% CO_2_) following intoxication. The cells were then harvested by scraping in OPP-containing medium and transferred into 1.5 mL reaction tubes. After centrifugation (500 rcf, 3 min), the supernatant was discarded, and the pellet was washed once with 1% BSA in PBS (wash step). This was followed by fixation of the cells for 15 min in 4% paraformaldehyde (Merck) at RT, a washing step, and permeabilization in a saponin-containing permeabilization buffer (0.1% saponin, 0.5% BSA, and 0.01 NaN_3_ in PBS) for 15 min at RT. After centrifugation (500 rcf, 3 min), the cells were stained in 100 µL reaction cocktail (prepared according to the manufacturer’s instructions; the cocktail includes the green-fluorescent AZDye 488 Azide Plus) for 20 min in the dark at RT. Finally, the cells were washed twice and resuspended in PBS for analysis. Green fluorescence, indicative of OPP incorporation, was measured using a BD FACSCelesta flow cytometer (488 nm blue laser; 530/30 filter). Flow cytometry data were analyzed using Flowing Software (Turku Bioscience Centre). Doublets were excluded via FSC-A/FSC-H gating, and green fluorescence histograms were used to distinguish OPP-positive from OPP-negative cells and to quantify respective cell populations.

### Cell surface-binding assay

For binding studies, the fluorescently labeled non-toxic DT mutant His-eGFP-CRM197 was used. Confluent HeLa cells were detached with 25 mM EDTA solution in PBS for 20 min (37°C, 5% CO_2_) and subsequently washed off the plate in PBS. 2 × 10⁵ cells were transferred to pre-blocked (culture medium) reaction tubes, centrifuged at 500 rcf for 3 min, and kept on ice from this point to prevent endocytosis. The cell pellet was resuspended in ice-cooled serum-free medium with or without 40 µM MLN and incubated for 20 min, followed by the addition of 250 nM His-eGFP-CRM197 and further incubation for 15 min. Finally, the cells were washed once (500 rcf, 3 min) and resuspended in PBS for analysis. Green fluorescence was measured using a BD FACSCelesta flow cytometer (488 nm blue laser; 530/30 filter). Analysis was performed with the Flowing Software (Turku Bioscience Centre). Doublets were excluded via FSC-A/FSC-H gating, and green fluorescence histograms were used to determine the median fluorescence intensity of the cells.

### Endosomal acidification assay

The effects of MLN4924 on endosomal acidification in living HeLa cells were assessed by fluorescence microscopy. Therefore, 0.18 × 10⁵ cells/well were seeded in a µ-Slide eight-well plate and incubated for 24 h (37°C; 5% CO_2_). The cells were then treated either with 40 µM MLN4924 for 20 min or with 200 nM Bafilomycin A1 for 1 h (37°C; 5% CO_2_). After two washing steps with PBS, the cell nuclei were stained with Hoechst33342 (1:10,000, ThermoFisher) and the acidified cell compartments with 50 nM LysoTracker Green DND-26 (Invitrogen) for 5 min (37°C; 5% CO_2_). After a further washing step, the slides were examined via fluorescence microscopy using the Keyence fluorescence microscope BZ-X810 with a Plan Apochromat 40× objective and the GFP (green, LysoTracker) and DAPI (blue, Hoechst33342) filter cubes (Keyence Deutschland GmbH, Neu-Isenburg, Germany).

### Immunoblot analysis of the neddylation status in MLN4924- and arctigenin-treated HeLa cells

To assess the impact of the neddylation inhibitors MLN4924 and arctigenin, respectively, on the neddylation status of Cullin-1 (Cul1) which is the main target protein for neddylation by NEDD8, HeLa cells (1 × 10^5^ cells/well) were first seeded in 24-well plates for 24 h (37°C, 5% CO_2_). Subsequently, the cells were treated with fresh culture medium containing MLN4924 or arctigenin, as indicated, for 20 min or 24 h (37°C, 5% CO_2_). For whole-cell lysate preparation, the cells were washed with PBS and harvested by scraping in pre-heated Laemmli buffer (0.3 M Tris-HCl, 10% SDS, 37.5% glycerol, 0.4 mM bromophenol blue, and 100 mM DTT). Lysates were further heated at 95°C for 10 min, resolved by SDS-PAGE using Bolt 4–12% Bis-Tris Plus WedgeWell gradient gels, and transferred onto a nitrocellulose membrane. Neddylated Cul1, which migrates at a higher molecular weight than non-neddylated Cul1, was detected using a polyclonal anti-Cul1 primary antibody (1:500, ThermoFisher Scientific, USA) and a mouse-anti-rabbit IgG-HRP (1:2,500, Santa Cruz Biotechnology, USA). β-actin served as loading control and was detected with a CoraLite 594-conjugated monoclonal anti-β-actin antibody (1:1,000, Proteintech, Germany).

### Enzyme activity assay of DTA in HeLa lysates

For the investigation of the *in vitro* enzymatic activity of DTA, HeLa lysates with non-denatured proteins were generated. Therefore, HeLa cells were seeded in 10 cm culture dishes until they reached a confluence of ~80%. Next, the cells were washed with PBS and lysed by a freeze-thaw cycle without buffer at −20°C for 20 min. ADP-ribosylation buffer (20 mM Tris-HCl, 1 mM EDTA, 1 mM DTT, 5 mM MgCl_2,_ 1:50 c0mplete) was added and the lysates were collected in reaction tubes. After centrifugation at 10,000 rcf for 1 min to remove cell debris, the supernatant was collected, and total protein concentration was measured using a Nanodrop spectrophotometer. 30 µg of the cell lysate were mixed with 40 µM MLN4924 and ADP-ribosylation buffer to get a total sample volume of 20 µL. For ADP-ribosylation and biotin-labeling of elongation factor 2 (EF2), 5 pM DTA and 1 µM biotin-labeled NAD^+^ (R&D Systems) were added to the lysate. After incubation at 37°C for 30 min, the samples were denatured and reduced in Laemmli buffer (0.3 M Tris-HCl, 10% SDS, 37.5% glycerol, 0.4 mM bromophenol blue, 100 mM DTT) at 95°C for 10 min. Next, lysate proteins were resolved by SDS-PAGE and transferred to a nitrocellulose membrane. Streptavidin-peroxidase (Sigma-Aldrich, Germany) was used for the detection of ADP-ribosylated, biotinylated EF2. GAPDH levels were detected with a primary GAPDH antibody (1:1000, Santa Cruz Biotechnology, USA) for assaying equal loading of the samples.

### Membrane translocation assay

The membrane translocation assay was employed to model and investigate the pH-dependent translocation of DT across the endosomal membrane at the plasma membrane. Therefore, HeLa cells (8 × 10^4^ cells/well) were seeded in 12-well plates (37°C, 5% CO_2_). After 48 h, the cells were pretreated with bafilomycin A1 (BafA1), an established inhibitor of endosomal acidification, in the presence or absence of MLN4924, as indicated for 20 min (37°C, 5% CO_2_). The cell culture plates were then transferred on ice and incubated for 5 min to cool down the cells for inhibiting endocytosis. A pre-nicked form of DT (nDT) was then added to the cells and incubated for 30 min on ice to allow surface binding. Unbound nDT was removed by washing the cells with PBS. Translocation of the enzyme subunit of nDT across the plasma membrane was triggered by incubating the cells in acidic, serum-free medium (pH 4) for 10 min at 37 °C. Cells treated with neutral medium (pH 7.4) served as negative control. Subsequently, the acidic medium was replaced again by serum-containing culture medium with BafA1 and neutral pH. MLN4924 was present in a concentration of 40 µM throughout the experiment, where indicated. Morphological changes induced by the enzyme subunit of nDT were documented with a Leica MC170 HD camera (Leica Microsystems Ltd.) connected to Leica DMi1 light microscope (Leica Microsystems CMS GmbH). The percentage of rounded cells out of the total amount of cells in each image was quantified manually.

### Statistics

If not stated otherwise, all experiments were conducted independently at least three times. The number of replicates (*n*) for each experiment or condition is indicated in the respective figure legends. Statistical analyses were performed using one-way ANOVA followed by Dunnett’s multiple comparison test, implemented in GraphPad Prism version 10 (GraphPad Software Inc., San Diego, CA, USA). The obtained *P* values are depicted as follows: ns = not significant, *P* > 0.05, *P < 0.05, **P < 0.01, ***P < 0.001, and ****P < 0.0001.

## SUPPLEMENTAL MATERIAL

Supplemental figures spectrum.03872-25-s0001.pdf Figures S1 to S4.

ASM does not own the copyrights to Supplemental Material that may be linked to, or accessed through, an article. The authors have granted ASM a non-exclusive, world-wide license to publish the Supplemental Material files. Please contact the corresponding author directly for reuse.
