# Supplementary material for: Pharmacological inhibition of host cell neddylation reduces intoxication of cells by diphtheria toxin and clostridial enterotoxins TcdB and C2
Source: Microbiol Spectr. 2026 Apr 2;14(5):e03872-25. doi: 10.1128/spectrum.03872-25 (PMC13141968; doi:10.1128/spectrum.03872-25)
Supplement: Supplemental figures — Figures S1 to S4. [file spectrum.03872-25-s0001.pdf]

Supplemental material – König et al.

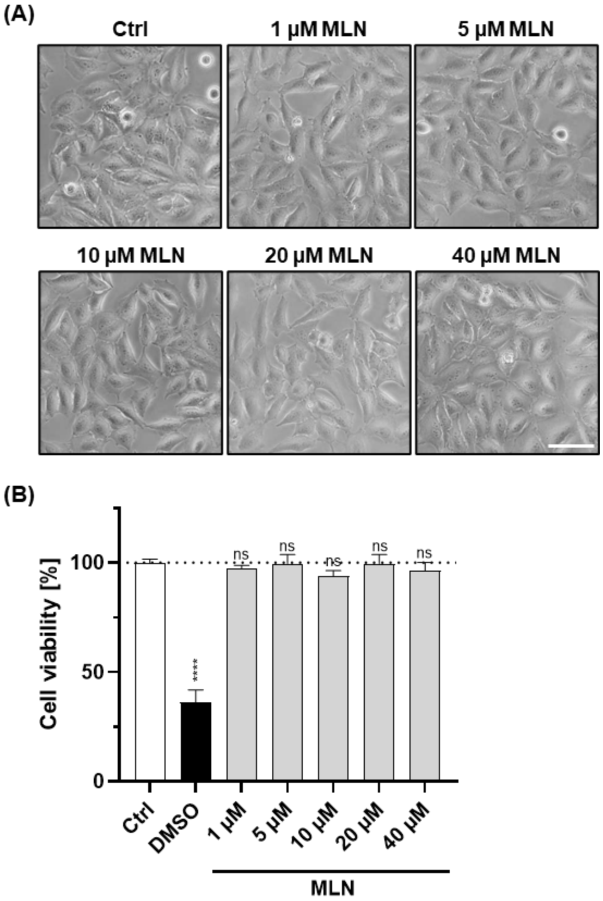

Figure S1: Effect of MLN4924 on morphology and viability of HeLa cells. Cells were incubated with MLN4924 (MLN) in increasing concentrations as indicated. (A) Representative microscopic images after 5 h are shown. (B) Cell viability was assessed 5 h after MLN4924 addition via MTS assay. Untreated (Ctrl) and 20% DMSO treated cells (DMSO) served as controls. Scale bar (white) represents 100 µm. Values are given as mean ± SEM of three independent assays with triplicates (n=9). Statistical significance between untreated cells (Ctrl) and DMSO-/ MLN4924-treated cells was determined (ns > 0.05; ****p < 0.0001).

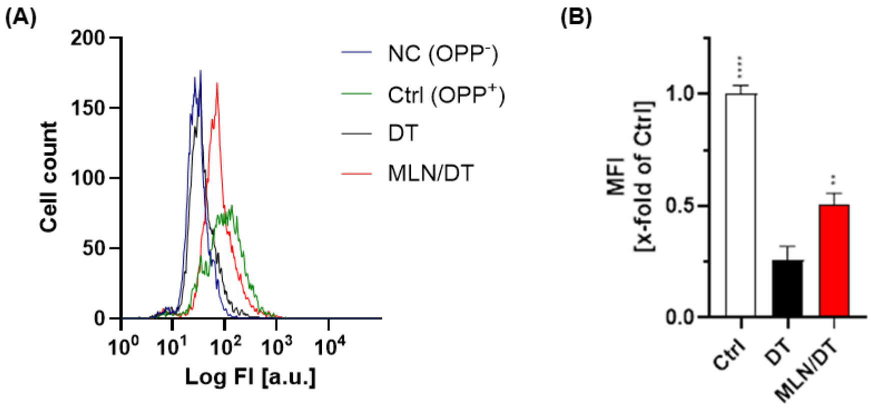

Figure S2: Effect of MLN4924 on protein synthesis in DT-treated Vero cells. Flow cytometry-based analysis of OPP incorporation in cells following DT intoxication. (A) Representative histogram of all conditions is shown. Cells were treated as indicated either with 2 nM DT (DT), the combination of 80 µM MLN4924 and DT (MLN/DT) or were left untreated (Ctrl (OPP+)). The conditions containing MLN4924 were preincubated for 20 min at 37 °C with the component prior to DT addition. 90 min after intoxication, 30 µM OPP were added to all samples except for a negative control (NC (OPP-)) representing autofluorescence of the cells. Each measurement was performed with 10.000 cells. (B) Quantitative analysis of (A). Shown is the median fluorescence intensity of cells x-fold to the untreated control (Ctrl), which was set to 1. Values are given as mean ± SEM of three independent assays with triplicates (n=9). Significance was determined between the DT only condition (DT) and all other conditions (**p < 0.01, ****p < 0.0001).

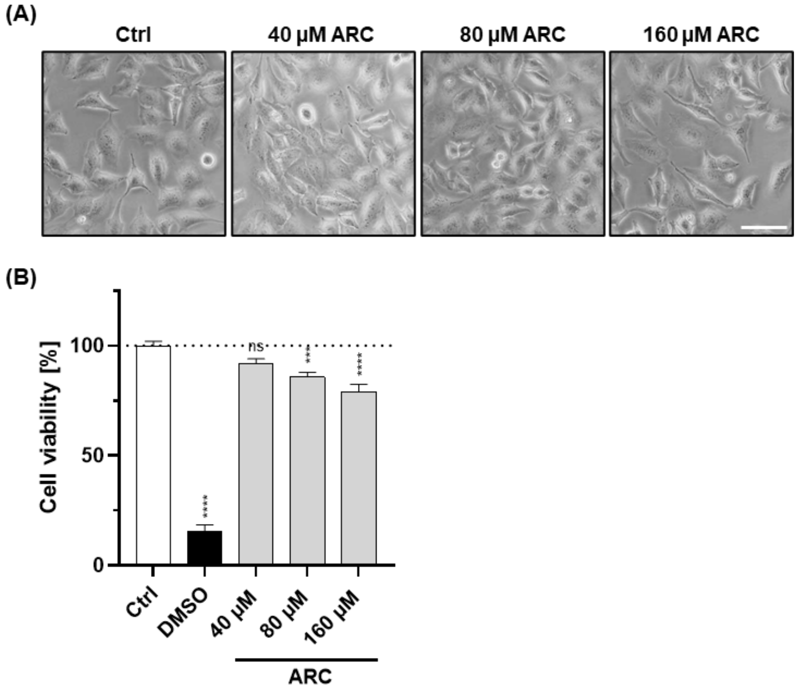

Figure S3: Effect of arctigenin on morphology and viability of HeLa cells. Cells were incubated with increasing concentrations of arctigenin (ARC) as indicated. (A) Representative microscopic images from cells after 5 h are shown. Scale bar (white) represents 100 µm. (B) Cell viability was assessed 5 h after ARC addition via MTS assay. Untreated (Ctrl) and cells treated with 20% DMSO (DMSO) served as controls. Values are given as mean ± SEM of four independent assays with triplicates (n=12). Statistical significance between untreated cells (Ctrl) and DMSO-/ ARC-treated cells was determined (ns > 0.05; ***p < 0.001; ****p < 0.0001).

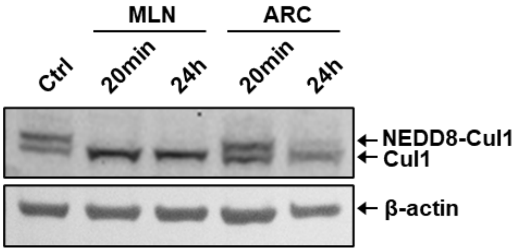

Figure S4: Effect of MLN4924 and arctigenin on Cullin-1 neddylation in HeLa cells. Cells were incubated with 40 µM MLN4924 (MLN) or 160 µM arctigenin (ARC) for 20 min or 24 h and lysed in Laemmli buffer with DTT. Untreated cells served as control (Ctrl). The whole-cell lysates were applied to SDS-PAGE and Cullin-1 was detected by Western blotting. The loss of the upper band of Cullin-1 (NEDD8-Cul1) indicates an inhibition of protein neddylation by both inhibitors. ß-actin was detected for loading control.
